# GastroGPT: Development and controlled testing of a proof-of-concept customized clinical language model

**DOI:** 10.1055/a-2637-2163

**Published:** 2025-08-06

**Authors:** Cem Simsek, Mete Ucdal, Enrique de-Madaria, Alanna Ebigbo, Petr Vanek, Omar Elshaarawy, Theodor Alexandru Voiosu, Giulio Antonelli, Román Turró, Javier P Gisbert, Olga P. Nyssen, Cesare Hassan, Helmut Messmann, Rajiv Jalan

**Affiliations:** 1 1501 Gastroenterology & Hepatology, Johns Hopkins Medical Institutions Campus, Baltimore, United States; 2 64005 internal medicine, Hacettepe University Faculty of Medicine, Ankara, Turkey; 3 16802 Dr Balmis General University Hospital, Alicante, Spain; 4 39694 Division of Gastroenterology, Universitätsklinikum Augsburg, Augsburg, Germany; 5 48207 Palacky University Olomouc, Olomouc, Czech Republic; 6 4595 Liverpool University Hospitals NHS Foundation Trust, Liverpool, United Kingdom of Great Britain and Northern Ireland; 7 68873 National Liver Institute, Shebeen El-Kom, Egypt; 8 Gastroenterology, Colentina Hospital, Bucharest, Romania; 9 117698 Sapienza University of Rome, Digestive and Liver Disease Unit, Azienda Ospedaliera Sant'Andrea, Roma, Italy; 10 Endoscopy Unit,, Teknon Medical Center, Barcelona, Spain; 11 16517 Division of Gastroenterology, Faculty of Medicine, Hospital Universitario de la Princesa, Madrid, Spain; 12 16517 Hospital Universitario de la Princesa, Madrid, Spain; 13 551905 Digestive Endoscopy Unit, Humanitas Research Hospital Department of Gastroenterology, Milan, Italy; 14 9687 University College Hospital London Medical School, London, United Kingdom of Great Britain and Northern Ireland

**Keywords:** Endoscopy Upper GI Tract, Reflux disease, Endoscopy Small Bowel, Inflammatory bowel disease, Neoplasia, Non-variceal bleeding, Pancreatobiliary (ERCP/PTCD)

## Abstract

**Background and study aims:**

Current general-purpose artificial intelligence (AI) large language models (LLMs) demonstrate limited efficacy in clinical medicine, often constrained to question-answering, documentation, and literature summarization roles. We developed GastroGPT, a proof-of-concept specialty-specific, multi-task, clinical LLM, and evaluated its performance against leading general-purpose LLMs across key gastroenterology tasks and diverse case scenarios.

**Methods:**

In this structured analysis, GastroGPT was compared with three state-of-the-art general-purpose LLMs (LLM-A: GPT-4, LLM-B: Bard, LLM-C: Claude). Models were assessed on seven clinical tasks and overall performance across 10 simulated gastroenterology cases varying in complexity, frequency, and patient demographics. Standardized prompts facilitated structured comparisons. A blinded expert panel rated model outputs per task on a 10-point Likert scale, judging clinical utility. Comprehensive statistical analyses were conducted.

**Results:**

A total of 2,240 expert ratings were obtained. GastroGPT achieved significantly higher mean overall scores (8.1 ± 1.8) compared with GPT-4 (5.2 ± 3.0), Bard (5.7 ± 3.3), and Claude (7.0 ± 2.7) (all *P* < 0.001). It outperformed comparators in six of seven tasks ( *P* < 0.05), except follow-up planning. GastroGPT demonstrated superior score consistency (variance 34.95) versus general models (97.4–260.35) ( *P* < 0.001). Its performance remained consistent across case complexities and frequencies, unlike the comparators ( *P* < 0.001). Multivariate analysis revealed that model type significantly predicted performance ( *P* < 0.001).

**Conclusions:**

This study pioneered development and comparison of a specialty-specific, clinically-oriented AI model to general-purpose LLMs. GastroGPT demonstrated superior utility overall and on key gastroenterology tasks, highlighting the potential for tailored, task-focused AI models in medicine.

## Introduction

Artificial intelligence (AI) has emerged as a potentially transformative technology in healthcare, demonstrating remarkable efficacy across various domains, including medical imaging and genomic analysis 1 2 3 4 . However, substantial challenges remain in translating early AI prototypes into impactful real-world tools that integrate safely and effectively into clinical workflows 5 . The global healthcare system's considerable strain exacerbates the need for advanced AI capabilities in medicine. Escalating service demands amidst provider shortages have led to overburdened staff, care delays, and exacerbated inequities in access 6 7 8 . For instance, appointment wait times for gastrointestinal specialty care average over 65 days in certain high-income regions 9 10 . Prolonged delays can result in disease progression, diminished productivity, and compromised quality of life 9 10 . Lengthy delays can result in disease progression, lost productivity, and reduced quality of life 11 12 . Enhancing efficiency and expanding the reach of specialized expertise could potentially address these gaps. AI-enabled solutions, such as customized clinical decision support systems, hold significant promise in augmenting provider workflows, optimizing patient triage, and increasing accessibility to high-value care 3 13 .

To date, most clinical applications utilizing natural language processing models have pursued generalist approaches, aiming for broad applicability. For example, the large language model (LLM) ChatGPT has demonstrated promise in select focused tasks, including medical exam performance, information retrieval from clinical notes, and patient counseling 14 15 16 17 . However, utilization of these models remained limited because they inherently lack customization to the intricate diagnostic and management considerations of specialized fields.

**Fig. 1:**
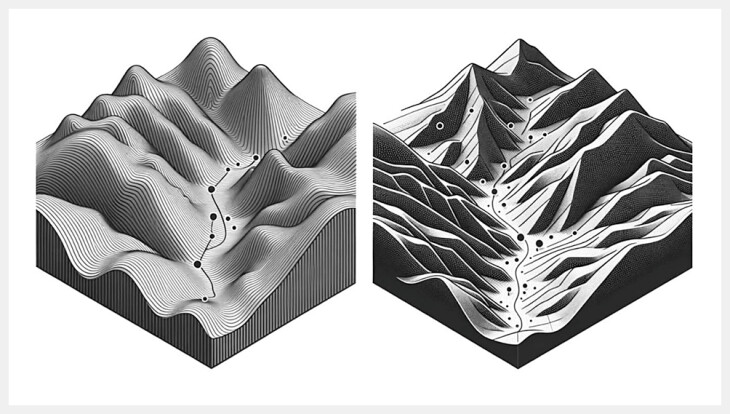
Topographic analogy of language learning models in general vs. clinical contexts. The left side of the figure represents general-purpose language learning models (LLMs) with a landscape of broad, undulating contours, symbolizing the extensive applicability and adaptability of these models across varied domains. The seamless transitions between elevations reflect the versatility and integrated problem-solving capabilities of general LLMs. In contrast, the right side illustrates clinical task-specific LLMs through a landscape of sharp ridges and narrow valleys, highlighting the models' focus and optimization for specialized clinical tasks. The steep, targeted pathways indicate the precision and depth of knowledge these models are designed to offer in healthcare settings. This topological representation serves as a visual metaphor for the operational divergence between LLMs designed for general use and those tailored for clinical applications, emphasizing their respective architectural and functional specializations.

We hypothesized that an AI system engineered specifically for the reasoning processes and goals of gastroenterology may better address this complexity ( Fig. 1 ). To test our hypothesis, we developed GastroGPT, a novel proof-of-concept clinical LLM purpose-built for gastroenterology using a transformer-based architecture. ChatGPT—an application built upon a Generative Pre-trained Transformer (GPT) neural network architecture—exemplifies a general text-generation framework that can be adapted for diverse applications. The term “GPT” refers to this broad, open-ended family of transformer-based models rather than any proprietary system. By contrast, GastroGPT extends these principles into the specialized domain of gastroenterology.

In this preliminary study, our central aim was to evaluate feasibility of a tailored LLM model and compare its performance with leading general-purpose AI systems. The key hypothesis was that customization would enhance utility for simulated gastroenterology tasks assessed by clinical experts.

## Methods

### Study design

This study employed a rigorous, blinded, controlled experimental design to evaluate feasibility and potential advantages of a specialized clinical AI system for gastroenterology. The novel system, GastroGPT, represents a customized transformer-based neural network architecture optimized specifically for clinical natural language processing in the field of gastroenterology. All data sources were free of protected health information in compliance with institutional policy, and per Institutional Review Board guidelines were deemed exempt from further ethical review due to prior anonymization and archival status. GastroGPT underwent comprehensive comparative analysis against three widely adopted general language models: GPT-4 (OpenAI, San Francisco, California, United States; coded as LLM-A), Bard (Google, Mountain View, California, United States; coded as LLM-B), and Claude (Anthropic, San Francisco, California, United States; coded as LLM-C).

### GastroGPT architecture and model development

The technical architecture of GastroGPT is predicated on a transformer-based neural network, enhanced by multi-head attention mechanisms and optimized for generating clinically accurate, contextually appropriate language pertinent to gastroenterology. The system utilizes a bidirectional encoder-decoder structure, wherein a deep encoder network encodes input text into high-dimensional vector representations, and a decoder generates relevant output text through iterative refinement. The model implements scaled dot-product attention and feed-forward neural networks in each layer to model complex linguistic context and capture specialized clinical terminology dependencies. The system employs advanced techniques including domain-specific fine-tuning, prompt engineering, and in-context learning, multiple semantic search algorithms for knowledge retrieval, chained inference mechanisms for logical reasoning, and adversarial training for robustness against out-of-distribution inputs.

GastroGPT's development centered on a curated training corpus comprising 1.2 million tokens, nearly 1500 pages of text, derived from gastroenterology-specific resources. The corpus integrated peer-reviewed publications from leading gastroenterology journals with conference materials, evidence-based clinical practice guidelines from international gastroenterology societies, and standardized reference textbooks. Data curation by a panel of four board-certified gastroenterologists (distinct from the 13 expert reviewers involved in the comparative evaluation), followed strict inclusion criteria overseen by five board-certified gastroenterologists, emphasizing current clinical evidence, procedural standards, and therapeutic protocols specific to gastroenterology. The training dataset also incorporated 10,000 clinical synthetic vignettes representing parts or complete cases with diverse gastroenterological presentations, management scenarios, and outcome patterns observed in tertiary care settings. Model development employed an iterative optimization approach utilizing gradient accumulation and mixed precision training to enhance computational efficiency while maintaining clinical accuracy. Performance validation occurred through systematic assessment of the model's diagnostic reasoning, therapeutic planning, and clinical decision-making capabilities across standardized test cases.

**Fig. 2:**
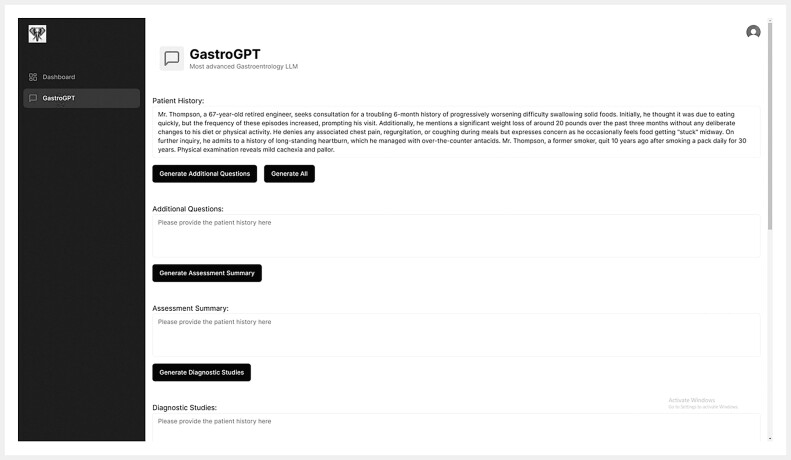
Screenshot of the interactive GastroGPT user interface (version utilized during this proof-of-concept study). The interface allows clinicians to enter patient histories, generate targeted additional questions, produce a concise assessment summary, propose relevant diagnostic investigations, propose medical management, program a follow-up schedule, recommend consultations, and finally create a patient information sheet. Button-driven workflows facilitate rapid navigation between clinical tasks, enabling streamlined data entry and retrieval of tailored gastroenterology recommendations.

A user-friendly web-based interface was designed for GastroGPT to streamline clinician interactions with the system. The interface enables users to enter patient histories, generate targeted additional questions, produce concise assessment summaries, propose relevant diagnostic investigations, suggest medical management plans, schedule follow-up visits, and recommend referrals as needed. The interface also has the functionality to compile this information into a patient-friendly information sheet. A screenshot showcasing the interactive features and button-driven workflow navigation is provided in Fig. 2 .

### Evaluation design

Power analysis was performed to determine the minimum number of scenario-level observations needed to detect a 1.0-point difference on a 10-point scale between GastroGPT and a comparator, with an assumed standard deviation of 1.2, a two-sided alpha of 0.05, and power of 0.90. These parameters indicated that 48 to 55 observations per model arm would be required. To accommodate up to 10% dropout or missing ratings and to enable more robust secondary analyses, we selected 10 simulated cases, each with eight tasks, yielding 80 scenario-level observations per model ( Table 1 , Table 2 ).

**Table:** **Table 1** Characteristics of simulated gastroenterology cases comprising the study cohort.

Case ID	Age	Case summary	Complexity	Frequency	Domain
1	67	Esophageal cancer	High	Uncommon	General gastroenterology
2	26	Appendicitis	Low	Common	Surgery
3	68	Hemorrhoids or fissure	Low	Common	General gastroenterology
4	30	Ulcerative colitis flare	Medium	Common	IBD
5	40	Chronic pancreatitis	Medium	Uncommon	Pancreas
6	43	GERD, fundoplication	Medium	Common	Endoscopy
7	61	Upper gastrointestinal bleed	Low	Common	General gastroenterology
8	55	Liver transplant, GVHD	High	Rare	Hepatology
9	16	Cyclic vomiting	High	Uncommon	Nutrition
10	46	Gastric lymphoma	High	Rare	Oncology
Cases represented a heterogeneous mix of patient demographics, clinical presentations, complexities, frequencies, and subspecialty domains to enable rigorous evaluation. GERD, gastroesophageal reflux disease; GVHD, graft versus host disease; IBD, inflammatory bowel disease.					

**Table:** **Table 2** Clinical workflow tasks and associated scoring rubric used by the expert physician panel to evaluate model outputs in a blinded fashion.

Number	Clinical task	Question	Score range
1	**Assessment and summary evaluation**	Does the summary accurately capture and emphasize relevant points from the case, guide care effectively, remain understandable, and maintain an optimum length?	1–10
2	**Additional history gathering**	Does the added history comprehensively cover relevant factors like Provocation, Quality, Region, Severity, Timing, risk factors, prior episodes, and medication, and align with clinical decision-making?	1–10
3	**Recommended studies**	Do the suggested studies match the differential diagnoses, benefit management, adhere to care standards, and utilize evidence-based medicine effectively?	1–10
4	**Proposed management**	Does the management plan adhere to treatment algorithms, offering comprehensive treatment options, and effectively weigh risks and benefits?	1–10
5	**Follow-up recommendations**	Are the follow-up plans and timings suitable for the patient's condition, in line with guidelines, and address long-term monitoring?	1–10
6	**Referral guidance**	Does the model aptly recommend specialist referrals and consider a multidisciplinary approach when needed?	1–10
7	**Patient counseling and communication**	Is the counseling patient-centric, promoting shared decision-making, and does it adapt explanations for varying health literacy levels?	1–10
8	**Overall quality of assessment**	How aligned are the model's recommendations with an expert's approach for the case?	1–10
The 10-point Likert scale assessed key dimensions including completeness, relevance, evidentiary basis, safety, practicality, and patient-centeredness.			

We initially created a bank of 100 simulated gastroenterology cases to represent a wide range of diseases and clinical complexities. To ensure feasibility and minimize reviewer burden, the required number of cases were randomly chosen from gastroenterology subspecialties for blinded assessment because each rater required approximately 3 to 4 hours to complete their evaluations.

Comparative assessment focused on seven key clinical tasks typical of gastroenterology practice: initial interpretation and summarization of the clinical vignette, elicitation of additional pertinent history, selection of appropriate diagnostic tests, development of an evidence-based management plan, determination of follow-up requirements, integration of specialty referrals and multidisciplinary care, and communication of findings and recommendations for patient counseling.

These tasks were selected to evaluate the models' capabilities across a spectrum of clinical activities performed by gastroenterologists. The first task assessed the models' ability to extract and synthesize relevant information from patient presentations. The second task evaluated the models' capacity to identify information gaps and formulate follow-up questions. The third task examined the models' knowledge of diagnostic protocols and their application. The fourth task assessed the models' capability to integrate clinical data with current practice guidelines. The fifth task evaluated the models' ability to create monitoring and reassessment plans. The sixth task examined the models' understanding of when to involve other specialists. The final task assessed the models' ability to communicate medical information in an accessible manner. This set of tasks was designed to provide a comprehensive assessment of the AI models' performance across key areas of clinical practice in gastroenterology.

**Fig. 3:**
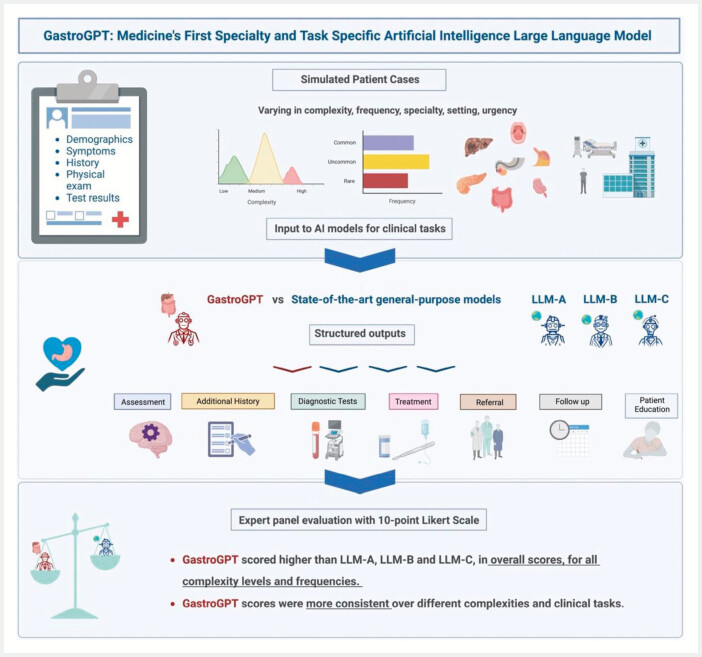
Visual overview of the study methodology, including the simulated patient cases, AI model comparison, clinical tasks evaluated, and expert panel evaluation process.

Model-generated outputs underwent evaluation by a diverse panel of expert physicians (n = 15) board-certified in gastroenterology, including those with subspecialty expertise. This multidisciplinary group assessed clinical sensibility and utility of model outputs in a double-blind fashion. A structured rubric was developed and validated to quantitatively grade the quality of model outputs across seven clinical task domains on a 10-point Likert scale. Fig. 3 provides a visual overview of the study methodology, including the simulated patient cases, AI model comparison, clinical tasks evaluated, and the expert panel evaluation process.

Fig. 3 illustrates the comparative performance of GastroGPT and general AI models across the seven clinical tasks evaluated in this study. The radar chart provides a visual representation of the mean scores achieved by each model for tasks including assessment and summary, additional history gathering, recommended studies, proposed management, follow-up recommendations, referral guidance, and patient counseling and communication.

### Statistical analysis

Statistical analyses were conducted using R (version 4.1.0) and Python (version 3.9) platforms. Analytical methodology encompassed verification of score distribution normality, descriptive statistical analyses, quantification of interrater reliability, primary comparative analysis between GastroGPT and general language models, subgroup analyses, assessment of homogeneity of variances, multivariable mixed-effects modeling, and sensitivity analyses. Additional analytical procedures included thematic analysis of qualitative feedback, application of the Benjamini-Hochberg procedure for controlling false discovery rate in multiple comparisons, and calculation of effect sizes.

This comprehensive methodological approach aimed to provide a thorough, objective evaluation of GastroGPT's performance in comparison with general-purpose AI models within the specific context of gastroenterology. The study's robust design, multi-faceted evaluation protocol, and rigorous statistical analysis offer valuable insights into the potential of specialty-specific AI systems in medicine, with significant implications for future development, validation, and clinical implementation of such technologies across various medical specialties.

## Results

The study design incorporated 10 simulated cases, four AI models (GastroGPT and three general-purpose LLMs), and 13 expert reviewers. The expert panel consisted of 13 gastroenterology attendings from academic institutions, with a mean experience of 16.5 years (range 3–40 years). These experts evaluated model outputs across seven clinical tasks using a standardized 10-point scale. Interrater reliability was robust, with an intraclass correlation coefficient of 0.89 (95% CI 0.87–0.91), indicating strong agreement among reviewers.

**Fig. 4:**
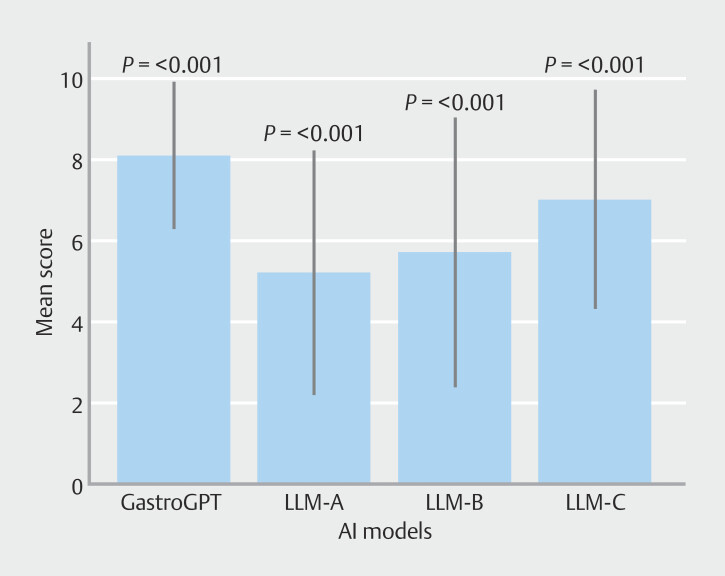
Radar chart depicting performance of GastroGPT and general AI models across the seven clinical tasks, with each axis representing a different task and distance from the center indicating mean score for that task.

GastroGPT demonstrated statistically significant superior performance (mean score 8.1 ± 1.8) compared with general AI models LLM-A (5.2 ± 3.0), LLM-B (5.7 ± 3.3), and LLM-C (7.0 ± 2.7) ( *P* < 0.001 for all comparisons). Paired comparisons revealed that GastroGPT significantly outperformed all general models, with a +3.0 point mean score advantage compared with LLM-A (95% confidence interval [CI] 2.7–3.2, t = 20.0, *P* < 0.001), a +2.4 point mean score benefit versus LLM-B (95% CI 2.1–2.7, t = 15.1, *P* < 0.001), and a +1.2 point mean score improvement relative to LLM-C (95% CI 0.9–1.4, t = 8.2, *P* < 0.001). Fig. 4 illustrates the overall performance comparison between GastroGPT and the general AI models ( Table 3 ).

**Table:** **Table 3** Comparison of mean evaluation scores between GastroGPT and general language models for each clinical task.

	GastroGPT	LLM-A	LLM-B	LLM-C	P
Assessment and summary evaluation	7.91 ± 1.70 ^*†^	6.09 ± 2.58	6.86 ± 2.13	7.89 ± 1.71	< 0.001
Additional history gathering	8.43 ± 1.83 ^*†‡^	2.98 ± 3.05	2.87 ± 3.23	2.84 ± 2.98	< 0.001
Recommended studies	7.90 ± 1.77 ^*^	4.50 ± 3.33	7.19 ± 2.85	7.36 ± 1.82	< 0.001
Proposed management	7.97 ± 2.09 ^*‡^	6.53 ± 2.17	7.73 ± 2.11	6.43 ± 2.97	< 0.001
Follow-up recommendation	7.51 ± 2.00 ^*†^	5.76 ± 2.53	4.33 ± 3.49	7.84 ± 1.98 ^*†^	< 0.001
Referral guidance	8.30 ± 1.67 ^*†‡^	4.57 ± 3.40	5.34 ± 3.48	7.77 ± 1.66	< 0.001
Patient counseling and communication	8.50 ± 1.73 ^*†‡^	5.09 ± 2.82	4.91 ± 3.30	7.87 ± 1.81	< 0.001
Overall quality of assessment	8.34 ± 1.29 ^*†‡^	5.84 ± 2.03	6.46 ± 2.13	7.89 ± 1.40	< 0.001
GastroGPT achieved statistically significantly higher scores than one or more comparators in all tasks. It outperformed all models in gathering pertinent history, integrating specialty referrals, and patient counseling. *P < 0.05 for LLM-A. ^†^ *P* < 0.05 for LLM-B. ^‡^ *P* < 0.05 for LLM-C. LLM-A, GPT4; LLM-B, Bard; LLM-C, Claude. Highest mean scores in each task are in boldface.					

**Fig. 5:**
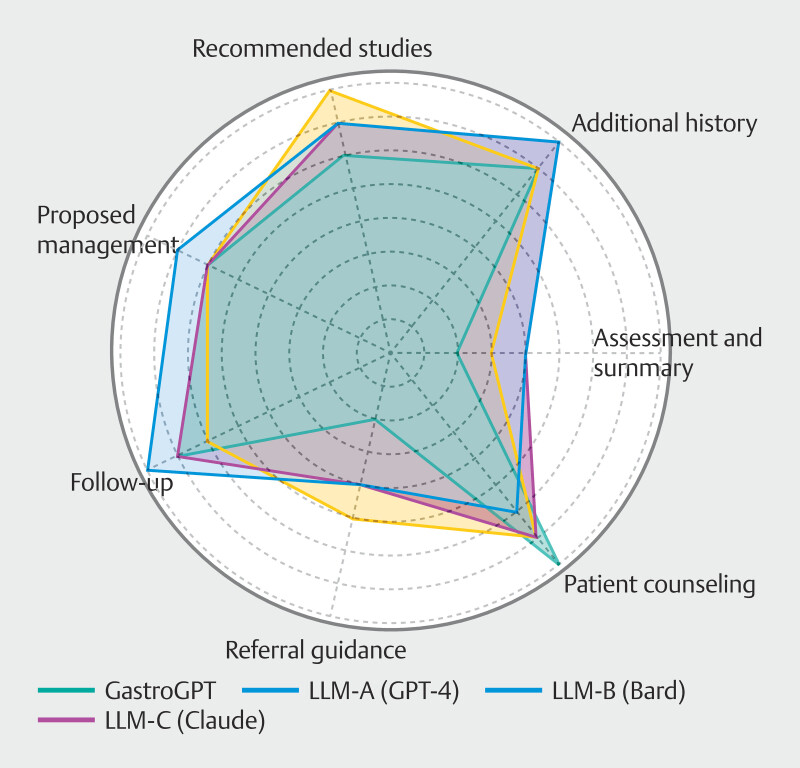
Radar chart visually demonstrating that GastroGPT consistently outperforms general AI models (GPT-4, Bard, and Claude) across most clinical tasks in gastroenterology, with its green area extending further from the center on nearly all axes, although some general models show competitive performance in specific tasks such as follow-up recommendations.

Analysis of the seven individual clinical tasks revealed that GastroGPT achieved higher mean scores than the general AI models across six of the seven tasks. GastroGPT significantly outperformed all three general models in additional history gathering, referrals, and patient counseling ( *P* < 0.001). It outperformed two of the models in assessment/summary, follow-up, and management tasks, and surpassed one model in the diagnostic studies task. Fig. 5 presents a detailed breakdown of performance across the seven clinical tasks for all models.

GastroGPT exhibited significantly lower variance in scores (34.95) compared with general models (variance 97.4–260.35, *P* < 0.001), indicating more consistent performance across varied clinical scenarios. In subgroup analyses, GastroGPT maintained significantly higher scores across all complexity and frequency levels ( *P* < 0.01 for all). For highly complex cases, GastroGPT averaged 7.9 (± 1.8) versus 5.1 to 6.8 for comparators ( *P* < 0.001). For least frequent disorders, GastroGPT scored 8.0 (± 2.1) compared with 5.9 to 7.2 for general models ( *P* < 0.001).

Multivariate analysis for reviewer and case clustering confirmed that model type and clinical task significantly impacted scores ( *P* < 0.001). Controlling for confounders, GastroGPT strongly predicted superior scores versus general models (coefficient 1.56, *P* < 0.001).

Linear regression found no linear associations between increasing case complexity nor decreasing case frequency and scores for both GastroGPT ( *P* > 0.05) and general models ( *P* > 0.05). However, while GastroGPT's mean scores were not different across complexity and frequency levels ( *P* = 0.21), the general models had significantly different mean scores between complexity and frequency subgroups ( *P* < 0.05 for all).

**Fig. 6:**
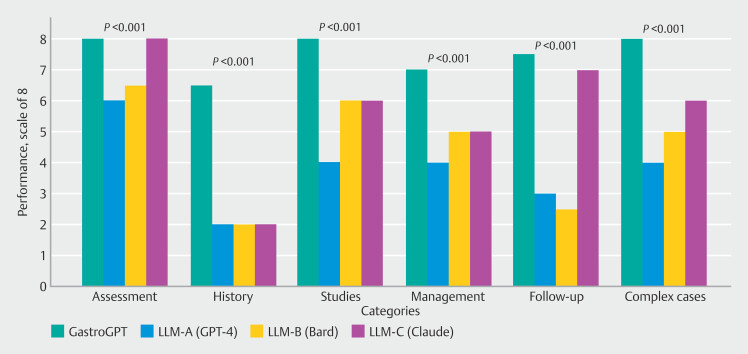
Bar chart demonstrating GastroGPT's superior performance across five clinical tasks and complex cases, with statistically significant differences ( *P* < 0.001) compared with general AI models.

It is noteworthy that LLM-B performed comparably to GastroGPT in follow-up planning without statistically significant difference ( *P* = 0.16), suggesting potential parity in this specific task. In addition, GastroGPT outperformed only one general model in diagnostic studies, indicating a potential area for further refinement and optimization. Fig. 6 presents a bar chart comparison of model performance across five key clinical tasks and complex cases, visually demonstrating GastroGPT's consistently superior scores and lower variance compared with general AI models.

## Discussion

This study introduced GastroGPT, a novel artificial intelligence model customized for gastroenterology, and compared its performance to leading general-purpose AI systems on simulated clinical cases. Blinded expert evaluation revealed that GastroGPT achieved statistically significantly higher scores across a range of gastroenterology workflow tasks, with a mean expert rating of 8.3 out of 10 compared with 5.6 to 7.8 for general models ( *P* < 0.001). GastroGPT outperformed comparators in six of seven specific clinical tasks, including patient assessments, diagnostic workups, management plans, referrals, and counseling.

These findings suggest that specialty-specific AI models like GastroGPT may offer significant advantages in clinical decision support within their domain of expertise. The consistent performance across varying case complexities and frequencies indicates potential for enhancing clinical workflows and decision-making processes in gastroenterology. However, the comparable performance in follow-up planning and limited superiority in diagnostic studies highlight areas for further investigation and potential improvement.

Analysis of individual tasks showed GastroGPT's particular strengths in additional history gathering and patient education, potentially reflecting its specialized design. However, in diagnostic testing and follow-up recommendations, the performance gap narrowed, suggesting areas for further refinement. The study's limited sample size precludes definitive conclusions about the mechanisms underlying these differences.

Notably, GastroGPT demonstrated more consistent performance across varying case complexities and frequencies compared with general models. This consistency may be attributed to specialized fine-tuning, which potentially allows for more focused attention on domain-specific knowledge.

These findings provide initial evidence that specialty-specific AI models may more effectively capture nuances of medical disciplines compared with generalized systems. However, larger-scale studies are needed to validate these results and explore their generalizability to real-world clinical settings.

Although GastroGPT generally exhibited superior performance, certain limitations warrant caution. First, biases may arise from the specialized dataset, which might overrepresent common or guideline-driven conditions while undersampling rarer gastroenterological presentations. Second, language models are susceptible to generating confidently stated inaccuracies—often termed “hallucinations”—when confronted with insufficiently represented scenarios. Third, our model may lag behind evolving clinical standards if not periodically retrained.

Recent studies have explored general models’ capabilities in various clinical tasks, with promising but mixed results. In diagnosis, ChatGPT demonstrated accuracy ranging from 42.7% on an ophthalmology exam 18 to 80% on microbiology questions 14 . For patient communication, one study found it generated more empathetic responses than physicians 19 , while another noted deficiencies in clinical reasoning 15 . Overall, ChatGPT shows aptitude for certain focused tasks like summarizing cases and counseling patients using natural language; however, limitations exist.

In clinical case interpretation, LLMs exhibit strengths and weaknesses. When summarizing gastroenterology cases, ChatGPT appropriately interpreted findings and made suitable recommendations in most scenarios 20 . However, deficiencies emerged in complex reasoning. On causal relationships for neuropathic pain diagnosis, ChatGPT struggled with consistency 21 . When advising on hypothetical infections, it lacked situational awareness and made inconsistent recommendations 15 . For patient communication, ChatGPT demonstrated an ability to interpret complex terminology and simplify explanations for diverse health literacy levels 20 . It also generated radiology report lay summaries that retained completeness without compromising safety 16 . However, appropriately adapting explanations for individual circumstances remains difficult ( 17 . In other clinical tasks, LLMs show utility in focused domains like summarizing cases or drafting clinical letters, but with known inaccuracies. For example, ChatGPT exhibits aptitude for discharge summaries and referral letters, but requires oversight to verify details 22 . It also shows potential for extracting information from patient notes, but outputs contain erroneous or fabricated details 23 . Overall, LLMs demonstrate capabilities that could improve efficiency for certain clinical tasks, but yet human validation is necessary to prevent errors.

Some studies have also begun gauging LLM performance relative to human clinicians. On United States Medical Licensing Examinations (USMLE)-style questions, GPT-3.5 scored similarly to passing medical students 24 . When advising on social media, ChatGPT responses were perceived as more empathetic than physicians' 19 . However, patients could not reliably distinguish LLM and human advice 25 . Comparisons remain preliminary, but ensuring safe and effective integration necessitates ongoing benchmarking against specialty experts. On overall medical licensing assessments, models have surpassed many benchmarks. On USMLEs, GPT-3.5 and GPT-4 attained scores comparable to senior medical students and residents 24 26 . However, specialty exam performance remains inconsistent. On an ophthalmology test, ChatGPT's accuracy varied across subspecialties 18 . Customized models like Med-PaLM 2, fine-tuned on medical data, have achieved expert-level performance, matching or exceeding GPT-4 in medical question-answering 27 .

In gastroenterology specifically, a study found that ChatGPT achieved passing scores on the 2021 and 2022 American College of Gastroenterology Self-Assessment (ACG-SA) exams when provided an engineered prompt to optimize its reasoning approach 28 . With this prompt engineering, ChatGPT scored 76% on the 2022 ACG-SA, matching average performance by human test-takers. It also showed accurate confidence self-assessments, correlating within 5% of actual accuracy. However, an earlier study using minimal prompt instructions found ChatGPT failed to pass these exams 29 . Prompt optimization appears crucial to maximizing clinical reasoning by general LLMs.

The modest performance gap with Claude points to the capabilities of general-purpose models in clinical tasks, because they are trained on diverse and vast data that also encompass medical resources. The task-specific performance results and differences between the models may show the dynamic interplay between model design, training, and task. GastroGPT’s limited advantage in the diagnosis task may be caused by its dependence on a broader medical knowledge and pattern recognition. For follow-up planning, Bard showed performance similar to GastroGPT ( *P* = 0.16), which may be a result of the task’s reliance on scheduling abilities rather than on clinical knowledge. For referral, GastroGPT performed better than other models, and this may be because other models do not have gastroenterology knowledge, but GastroGPT still falls short. For the assessment and summary task, GastroGPT performed better than ChatGPT and Bard but not Claude. This might be caused by Claude’s better language proficiency, which is a key asset in summarization tasks requiring concise and coherent synthesis. These results may show trade-offs between specialized and general-purpose models. The observed performance differences highlight how different tasks and model characteristics influence performance, also emphasizing the need for better evaluation metrics. In the future, combining domain-specific designs with general reasoning models could yield a better-performing hybrid model. Of course, prospective studies in real-world clinical settings are warranted to validate these findings and assess practical utility.

This study evaluated the efficacy of GastroGPT, a specialized LLM for gastroenterology, in comparison with general-purpose LLMs. Although general LLMs demonstrate proficiency in certain clinical tasks, they exhibit limitations in complex reasoning and situational adaptation. The research methodology employed a rigorous, blinded, comparative assessment using simulated cases evaluated by a multidisciplinary expert panel. Key strengths of the study include a controlled, reproducible head-to-head comparison, objective assessment through a blinded expert panel, comprehensive coverage of diverse clinical scenarios, and focus on real-world gastroenterology workflow tasks. The results indicate that GastroGPT outperformed general LLMs across multiple clinical tasks, suggesting potential benefits of specialty-specific AI models in capturing nuanced medical reasoning. However, the study has several limitations, including reliance on simulated cases necessitating validation with real-world data, potential for residual biases despite blinded evaluation, lack of direct comparison to human expert performance, and need for further refinement and expansion of the GastroGPT model.

The findings underscore the advantages of tailored AI systems for individual medical specialties over general conversational models. This study provides quantitative evidence supporting development of specialty-specific AI models to better address complex clinical tasks. Future research directions include larger-scale validation studies in real-world settings, optimization and expansion of tailored models across various medical specialties, and development of rigorous standards for clinical implementation. Another key limitation of our design is absence of a direct comparison between human clinician decisions and outputs produced by GastroGPT. Although the present study offers a controlled, blinded assessment against other language models, future investigations must evaluate how AI-driven recommendations align with—and potentially enhance—expert decision-making in actual clinical settings.

## Conclusions

In conclusion, although the results are promising, further research is required to confirm the efficacy, reliability, and ethical implementation of specialized clinical LLMs in real-world healthcare settings.
